# Trajectory of Iron and Red Cell Parameters in Moderately Anemic Iron-Deficient Pregnant Women Receiving Daily Iron–Folic Acid Supplementation: A Prospective Cohort Study

**DOI:** 10.3390/jcm14196787

**Published:** 2025-09-25

**Authors:** J. P. Akshaykirthan, Manjunath S. Somannavar, S. Yogeshkumar, M. S. Deepthy, Umesh Charantimath, Amaresh Patil, Mrutyunjaya B. Bellad, Richard Derman, Shivaprasad S. Goudar

**Affiliations:** 1 Department of Biochemistry, Jawaharlal Nehru Medical College, KLE Academy of Higher Education and Research, Belagavi 590010, Karnataka, India; akshay.kirthan.jp@gmail.com; 2 Department of Community Medicine, Jawaharlal Nehru Medical College, KLE Academy of Higher Education and Research, Belagavi 590010, Karnataka, India; yogeshkumar@jnmc.edu (S.Y.); drumesh.charantimath@gmail.com (U.C.); 3 Women’s & Children’s Health Research Unit, Jawaharlal Nehru Medical College, KLE Academy of Higher Education and Research, Belagavi 590010, Karnataka, India; deepthyms27@gmail.com (M.S.D.); amareshpatilp@jnmc.edu (A.P.); 4 Department of Obstetrics and Gynecology, Jawaharlal Nehru Medical College, KLE Academy of Higher Education and Research, Belagavi 590010, Karnataka, India; belladmb@gmail.com; 5 Department of Global Affairs, Thomas Jefferson University, Philadelphia, PA 19107, USA; richard.derman@jefferson.edu; 6 Department of Physiology, Jawaharlal Nehru Medical College, KLE Academy of Higher Education and Research, Belagavi 590010, Karnataka, India; sgoudar@jnmc.edu

**Keywords:** iron deficiency anaemia, oral iron supplementation, pregnancy, iron indices, red cell indices

## Abstract

**Background/Objectives**: The objective is to study the trajectories of hematologic and biochemical markers in moderately anemic pregnant women receiving oral iron supplementation throughout pregnancy. **Methods:** This prospective cohort study was conducted from August 2021 to September 2023 involving 315 pregnant women from rural areas of Belgaum, Karnataka, India, with hemoglobin levels between 7.0 and 9.9 g/dL and serum ferritin < 30 ng/mL and/or TSAT < 20%. Participants received iron–folic acid supplementation (IFAS) as per Anaemia Mukt Bharat guidelines. Blood samples were collected to measure various hematologic and iron markers and compared across each visits. **Results**: We report a complete adherence rate of 95.3% for iron and 97.8% for folic acid supplementation throughout pregnancy and also observed significant improvements in hemoglobin (9.36 (8.55, 9.74) to 12.03 (11.49, 12.72)) g/dL, hematocrit (29.93 ± 2.87 to 33.71 ± 3.69) %, MCV (72.16 ± 7.90 to 83.47 ± 7.65) fL, MCH (22.44 ± 3.01 to 26.77 ± 3.08) pg levels from the early second to the early third trimester of pregnancy with significant difference (<0.001). Increased erythropoiesis was reported by a higher reticulocyte hemoglobin (23.30 ± 3.03 to 27.84 ± 3.83) pg and immature reticulocyte fractions (6.90 (4.30, 9.50) to 7.30 (4.3, 11.0)) %. Initially, iron, ferritin and TSAT levels increased but later stabilized or slightly declined towards the end of pregnancy. **Conclusions**: Daily IFAS in moderately anemic pregnant women improved the trajectory of iron parameters, with peak gains in early third trimester. High adherence via counselling supports targeted monitoring and trimester-specific strategies to reduce maternal anemia and may improve outcomes.

## 1. Introduction

Iron deficiency anemia (IDA) is a prevalent and potentially serious condition that affects a significant number of pregnant women worldwide. It affects nearly 2 billion people worldwide, out of a global population of 7.5 billion, making it the most prevalent micronutrient deficiency [1]. A total of 52% of pregnant women in India are anemic, according to the NFHS-5, and, in Karnataka, the prevalence of anemia was reported to be 45.7% in pregnant women and 47.8% in non-pregnant women. The prevalence of anemia in Belgaum was found to be 44.1% among non-pregnant women and 37.3% among pregnant women [2]. During pregnancy, a woman’s body undergoes profound physiological changes, including an expansion of the mother’s blood volume and plasma to support the developing fetus [3]. A total of 3.46 milligrams of iron are needed for every gram of hemoglobin the mother synthesizes.

The fetus also needs iron to support metabolism, oxygen transport and relatively large accumulations of iron, which will be used during the first six months of postnatal life [4]. The placenta is an organ with a high iron requirement that is metabolically active. It can store iron in the reticuloendothelial cells. This provides a buffer during times when the mother’s iron supply is insufficient [5]. An additional gram of iron is required during pregnancy, which is distributed more evenly between mother and fetus. This increased demand for red blood cells and hemoglobin can strain the body’s iron reserves, which leaves pregnant women more susceptible to IDA. This can lead to maternal fatigue, weakness and a compromised immune system making the woman more susceptible to infections and illnesses. This can disrupt her ability to carry out daily activities and negatively impact her overall well-being. Therefore, it is crucial to understand the causes, risk factors and consequences of iron deficiency anemia in pregnancy as well as effective prevention and treatment strategies. The primary reasons for maintaining adequate iron levels throughout pregnancy are to safeguard the health of the mother, enhance the quality of the pregnancy and promote the development of the fetus.

Oral iron supplements are generally the first-line treatment for “iron deficiency” for most women [6]. It is recommended to take 60–120 mg of iron daily on an empty stomach in the morning to enhance absorption, preferably accompanied by ascorbic acid [7,8]. The previous literature suggests that the maternal indices improved after oral iron supplementation [9]. There is limited evidence on how oral iron supplementation in anemia affects the iron indices at specific time intervals during pregnancy. Therefore, the purpose of this study is to present a brief overview regarding oral iron treatment throughout pregnancy and to understand which phase of pregnancy could result in maximum changes in the iron indices. Hence, the objective of this paper is to study the trajectories of iron indices in moderately anemic pregnant women receiving oral iron supplementation.

## 2. Materials and Methods

### 2.1. Study Population

All pregnant women who visited Community Health Centre (CHC) and its Primary Health Centres (PHCs) in the rural areas of Belgaum, Karnataka, India, were screened at around 12–16 weeks of pregnancy. The inclusion criteria were as follows: pregnant women with gestational age of 12–16 weeks, age between 18 and 40 years, hemoglobin level between 7 and 9.9 g/dL (moderate anemia), and serum ferritin < 30 ng/mL and/or TSAT < 20%. Participants with twin pregnancy, any congenital anomaly, or anemia other than iron deficiency were excluded. Out of 367 eligible pregnant women, 315 women who consented to this study were enrolled using a consecutive sampling technique.

### 2.2. Sample Size

The sample size of 315 was determined by assuming a prevalence of 37.3% of anemia among pregnant women, a 95% confidence interval corresponding to 1.96 alpha, and the margin of error to be 15%, with 10% attrition [2].

### 2.3. Study Procedure

This prospective cohort study was conducted from August 2021 to September 2023 according to the guidelines of the Declaration of Helsinki, and all procedures involving humans were approved by the Institutional Ethical Committee of KLE Academy of Higher Education and Research (KAHER), Belgaum (KAHER/EC/21-22/001), nested within the RAPIDIRON Trial conducted at the Women’s and Children’s Health Research Unit of Jawaharlal Nehru Medical College, Karnataka [10]. Informed written consent was taken from every individual participating in this study in local languages (Kannada and Marathi). Blood samples were collected, and a questionnaire about sociodemographic characteristics with vital statistics and iron–folic acid compliance was also noted.

### 2.4. Treatment Dosage

All participants enrolled for this study were given single dose of deworming medication (Albendazole–400 mg) as per government guidelines and also received iron (60 mg of ferrous sulphate) and folic acid (400 mcg) tablets consistent with the most recent Anemia Mukt Bharat treatment guideline [11]. The administration of tablets for the treatment of anemia involved twice-a-day regimen. However, if the hemoglobin levels reached a normal range suitable for pregnancy by 26–30 weeks, specifically >11 g/dL, the tablets were then given at a prophylactic dose of once daily.

### 2.5. Analysis of Samples

Venous blood samples (2 mL in EDTA and 2 mL in a clot activator tube) were collected at multiple time points during pregnancy to assess the trajectories of blood iron indices. Sampling began at 12–16 weeks of gestation, followed by subsequent collections at 20–24 weeks, 26–30 weeks and 30–34 weeks with the final sample obtained on the day of delivery. After the sample collection, samples were transported to the tertiary care hospital laboratory by specimen transport box with ice packs ensuring the quality of samples is not compromised. The blood parameters include serum iron, transferrin saturation (TSAT), total iron binding capacity (TIBC), and ferritin and were immediately analyzed by Roche-Cobas-6000 (Roche Diagnostics, Basel, Switzerland). Hemoglobin, reticulocyte-hemoglobin (Ret-Hb), immature reticulocyte fraction (IRF) and complete blood count were analyzed in Sysmex-Hematology analyser. A subset of 105 samples of serum sTfR (soluble transferrin receptor) was also analyzed using a sandwich ELISA method.

### 2.6. Statistical Analysis

All the continuous variables were summarized using Mean/SD or Median (Q1, Q3) depending upon normality of the data. Kolmogorov–Smirnov test was used to assess the normality. All the categorical variables were summarized using frequency and percentage. Parameters with Two-time point were analyzed using paired *t*-test or Wilcoxon matched-pairs signed-rank test. One way Repeated Measures Analysis of Variance (RM-ANOVA)/Friedman’s nonparametric test was used to compare the average maternal indices at more than two time points. Post hoc analysis was carried out to adjust for Bonferroni corrections. Box plots were constructed to visualize the trends in the average maternal indices over the period. Imputation for missing data of continuous variables was conducted using a mean imputation method and assuming missing rate of 5–10%. All the statistical analyses were performed using R software version 4.2.3, and a *p*-value less than 5% was considered to be statistically significant.

## 3. Results

The baseline and socio-demographic characteristics of the 315 pregnant women who participated in this study are represented in Table 1. Most participants were between 18 and 25 years old (73.3%), with smaller proportions aged between 26 and 30 years (19.0%) and 31 years or older (7.6%). Maternal Hb at 12–16 weeks or recruitment showed a median Hb of 9.36 (Q1:Q3, 8.55:9.74). Based on body mass index (BMI), 33.7% of pregnant women were underweight, 58.1% had a normal weight, 7.0% were overweight and 1.3% of the pregnant women were classified as obese. Blood pressure readings showed a median systolic and diastolic blood pressure of 101 (Q1:Q3, 94:109) and 64 (Q1:Q3, 60:70) mm Hg, respectively. In total, 42.5% of the women were nulliparous, 28.6% were primiparous, and 28.9% were multiparous. Based on dietary habits, 7.3% were vegetarians, 14.9% were lacto-vegetarians, 17.1% were ovo-vegetarians and 60.6% predominantly ate a mixed diet.

Our study of 315 participants demonstrates a complete adherence (>75%) of 95.3% for iron and 97.8% to folic acid supplementation throughout pregnancy. Moderate adherence (50–75%) was observed in 15 (4.7%) of the pregnant women who used oral iron and 7 (2.2%) for ingestion of a folic acid tablet. A majority of pregnant women were compliant with oral and folic acid tablets. However, a small percentage of pregnant women, 17 (5.39%) and 11 (3.49%), were non-adherent to iron and folic acid, respectively.

The predominant reason for not taking iron tablets was that the tablets made them feel sick, reported by 14 women (4.44%). Similarly, 8 women (2.54%) reported the same for folic acid tablets. Two women (0.63%) reported forgetting to take both iron and folic acid tablets. Only one woman (0.32%) stated that she was unable to understand the instructions for taking both tablets. No participant reported losing or misplacing their tablets as a reason for non-compliance. These findings highlight the necessity of managing side effects and improving awareness to increase 100% adherence among pregnant women, which are depicted in Table 2.

Various side effects experienced by participants after taking iron tablets were also noted. In total, 118 of the 315 pregnant women reported side effects; of those, 43 (13.65%) indicated vomiting, 38 (12.06%) experienced nausea or stomach upset, 13 (4.13%) had diarrhea and 6 (1.90%) reported faintness. In addition, 15 (4.76%) experienced constipation, while 3 (0.95%) complained of vaginal bleeding.

### 3.1. Changes in Maternal Red Cell Indices During Pregnancy

The significant changes observed in red cell indices in the early second trimester and 26–30 weeks of pregnancy are represented in Table 3. Red blood cell (RBC) count decreased significantly from 4.19 million cells/µL at 12–16 weeks of GA to 4.05 million cells/µL at 26–30 weeks of GA (*p* < 0.001). Mean corpuscular volume (MCV) showed a notable increase from 72.16 to 83.47 fL. Similarly, mean corpuscular hemoglobin (MCH) rose from 22.44 to 26.77 pg, and mean corpuscular hemoglobin concentration (MCHC) rose from 31.03 to 32.01 g/dL between 12–16 weeks and 26–30 weeks of gestation, all changes statistically significant (*p* < 0.001). Red cell distribution width (RDW) percentage remained similar at both time points. Hematocrit (HCT) levels were higher at 26–30 weeks (33.71%) compared to 12–16 weeks (29.93%), and reticulocyte hemoglobin (Ret-Hb) assay showed a significant rise from 23.30 to 27.84 pg. Additionally, immature reticulocyte fraction (IRF) slightly increased from 6.90% to 7.30% over the same period, with all increases significant (*p* < 0.001). The most marked changes occurred in blood concentrations of MCV, MCH and in reticulocyte hemoglobin (Ret-Hb).

### 3.2. Trajectory or Trends of Maternal Iron Parameters During Pregnancy

**Figure 1:**
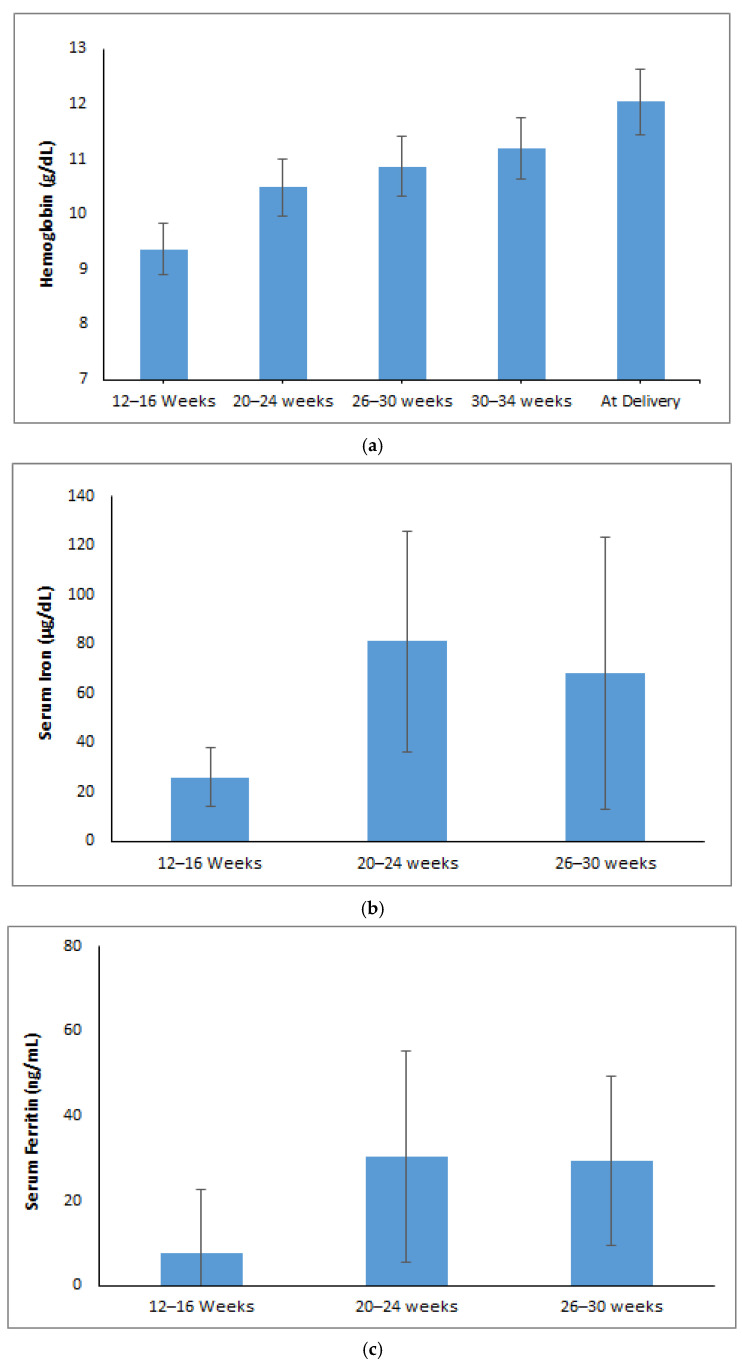

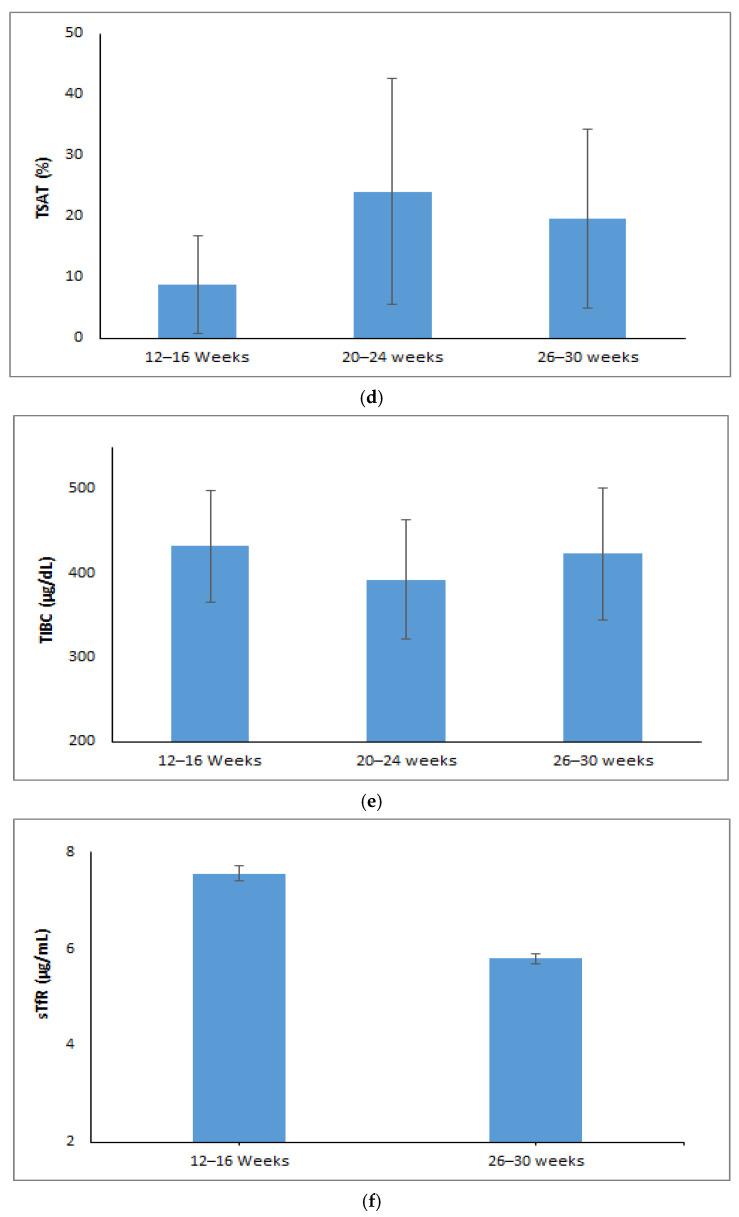
Trends of maternal iron parameters over different gestational age (weeks): (**a**) Hemoglobin (g/dL). (**b**) Serum iron (µg/dL). (**c**) Ferritin (ng/mL). (**d**) TSAT (%). (**e**) Serum TIBC (µg/dL). (**f**) sTfR (µg/mL).

The hemoglobin levels showed a notable increase from 9.36 g/dL at 12–16 weeks of gestational age (GA) to 12.03 g/dL at delivery (*p* < 0.001) (Figure 1a). Serum iron levels dropped to 68 µg/dL at 26–30 weeks of gestational age after increasing significantly from 26 µg/dL during the 12–16 week period to 81 µg/dL in the 20–24 week period (*p* < 0.001) (Figure 1b). The levels of serum ferritin increased significantly from 7.58 ng/mL at 12–16 weeks to 30.35 ng/mL at 20–24 weeks and then stabilized at 29.45 ng/mL at 26–30 weeks (*p* < 0.001) (Figure 1c). Transferrin saturation (TSAT) improved from 5.88% at 12–16 weeks to 20.58% at 20–24 weeks then slightly decreased to 16.58% at 26–30 weeks of GA (*p* < 0.001) (Figure 1d). Total iron-binding capacity (TIBC) decreased from 432 µg/dL at 12–16 weeks to 392 µg/dL at 20–24 weeks and then increased to 423 µg/dL at 26–30 weeks (*p* < 0.001) (Figure 1e). Soluble transferrin receptor (sTfR) levels significantly dropped from 7.55 µg/mL at 12–16 weeks to 5.79 µg/mL by 26–30 weeks of GA (*p* < 0.001) (Figure 1f).

Post hoc comparisons adjusted for Bonferroni corrections were performed for those maternal iron parameters (hemoglobin, iron, ferritin, TIBC, TSAT) that were statistically significant. The post hoc analysis revealed a statistically significant difference in the average hemoglobin, iron, TSAT between the gestational ages of 12–16 week and 20–24 week (*p* < 0.05), whereas TIBC showed significant differences across 20–24 and 26–30 weeks of gestation but showed no substantial difference between 12–16 weeks and 26–30 weeks (*p* = 1.00). Additionally, analysis for ferritin showed no significant difference at 20–24 weeks and 26–30 weeks of gestational age (*p* = 0.98) but did show a statistical difference across other gestational ages (*p* < 0.05).

## 4. Discussion

### 4.1. The Main Findings

We investigated the hematologic response to oral iron supplementation in moderate iron-deficient pregnant women and observed significant improvements in the concentrations of both hematologic and biochemical parameters during pregnancy. The current study reported an adherence rate of 95.3% for iron and 97.8% for folic acid supplementation throughout the pregnancy. Moderate adherence was observed in 4.7% of the pregnant women for iron and 2.2% for folic acid tablets. This high adherence is crucial for the effectiveness of supplementation programmes. A similar adherence rate has been reported in a study conducted in another region [12]. Although we did not examine the relationship between antenatal care visits and adherence to iron tablets, previous research has shown that attending four or more prenatal care visits is associated with improved adherence to iron supplements during pregnancy [13].

A study conducted in Northern Ghana reported an adherence rate of 84.5% for iron and folic acid supplementation (IFAS) among pregnant women [14]. An evaluation of adherence to IFAS among pregnant women at a tertiary care centre found that 63.8% of participants followed the recommended supplementation guidelines [15]. However, one study reported a much lower adherence rate of 28.7% for IFA tablets, adding that adherence rates were lower due to socioeconomic differences, data collection methods, healthcare professional training and varying healthcare institution standards across facilities [16]. These variations highlight the need to address region-specific barriers to improve adherence rates universally. Given that, side effects reported by participants (tablets made me feel sick) were the main reason for the small number of non-compliance with iron supplementation in this population. Encouraging them to take the pills with food may alleviate these symptoms [17]. Furthermore, forgetfulness is a barrier to iron supplementation for some women; it is very important to provide education during prenatal visits on strategies to assist study participants in taking their tablets. For instance, placing the tablets in a daily visible location, such as on a kitchen counter or night table, as advised by earlier authors, can be beneficial [18]. In accordance with Moretti et al., who demonstrated that administering oral iron more than once daily does not enhance iron absorption, our study utilized a twice-daily regimen [19]. The compliance rate observed was so high that it remains uncertain whether once-daily iron administration would have produced a significantly greater hematological response, but it is plausible that such a regimen might have reduced gastrointestinal side effects, which were the main reason for noncompliance.

The improvements in maternal iron parameters observed in our study are consistent with findings from other studies. A steep increase in hemoglobin levels of participants was observed from the early second trimester to delivery. This is in line with the study by Fisher and Nemeth, who reported significant increases in hemoglobin levels following iron supplementation in pregnant women with IDA [3]. Our study findings support the evidence that iron supplementation effectively enhances maternal hematologic status. Our study documented that, in the early third trimester of pregnancy, there is a notable increase in hematocrit, hemoglobin concentration and other red cell indices. This rise can be linked to a reduced expansion of plasma volume and is particularly evident in pregnant women who use iron supplements. A study has shown that pregnant women who take iron supplementation tend to have elevated levels of hematocrit, hemoglobin concentration, and red cell count compared to those who do not consume iron supplements. These findings emphasize the importance of considering iron intake for maternal health during pregnancy [20].

We found that MCV, MCH and MCHC values were low in the early second trimester and observed a significant increase during the early third trimester. Similarly, a gradual rise in MCV and MCH from early pregnancy until delivery was observed in another study [21]. The expansion of plasma volume and higher nutritional demands are the main causes of the initial lower level of MCV, MCH and MCHC values during the early first trimester. These parameters rise as pregnancy progresses into the early third trimester (enhanced erythropoiesis, iron supplementation, and other physiological adaptations) in order to support the developing demands of the fetus and get the mother’s body ready for delivery [22]. This study found that RDW (%) remained stable during pregnancy, aligning with a study on Sudanese pregnant women, which reported RDW as less sensitive to iron status changes than serum ferritin [23]. The study also noted no significant variations in MCV, MCH and RDW as these parameters reflect averages and may not capture minor cell size changes especially in early iron deficiency. A Cochrane review suggested that the lack of RDW response to oral iron supplementation (daily or intermittent) may result from the body’s homeostatic regulation of red cell production during pregnancy [24].

IRF and Ret-Hb are newer, sensitive measures of erythropoiesis [25]. IRF reflects bone marrow activity but does not indicate iron incorporation into developing RBCs. In cases like bleeding, IRF may increase without a corresponding rise in hemoglobin [26]. Ret-Hb, on the other hand, measures hemoglobin content in newly produced RBCs, serving as a real-time indicator of iron availability for erythropoiesis. Unlike acute-phase reactants, Ret-Hb is effective in detecting responses to iron therapy and is a more reliable measure of iron supply [27]. Our study showed a drastic increase in Ret-Hb and IRF levels from baseline to the early third trimester. Use of Ret-Hb as a parameter to predict early response to exclusive oral iron therapy in children revealed that the mean Ret-Hb before treatment was 18.20 pg. After iron supplementation, it increased to 21.48 pg and then to 25.43 pg at two different time points. The researchers concluded that an increase in Ret-Hb implies that iron supplementation improves erythropoiesis [28]. In our study we found an initial rise in median iron, ferritin, and TSAT from baseline to 20–24 weeks of gestation. Ferritin remained stable at 26–30 weeks, while iron and TSAT decreased to a smaller extent. TIBC slightly decreased during 20–24 weeks and rose significantly during 26–30 weeks. In contrast with our findings, a study reported that ferritin, iron, and TSAT levels decreased, and TIBC increased from early to late pregnancy [29]. There was an initial increase in serum iron and TSAT with daily iron supplementation; these levels plateaued or slightly decreased in the later stages of pregnancy [7].

A study revealed that hemoglobin concentration, serum iron, ferritin levels and red blood cell count all experienced a significant decline in the third trimester of pregnancy compared to the first and second trimesters. On the other hand, compared to the first and second trimesters, the third trimester had significantly greater levels of TIBC, transferrin and the sTfR: ferritin ratio [30]. It is established that serum sTfR levels are a sensitive and specific indicator of cellular iron need. Because it increases in situations where erythropoiesis is enhanced and tissue iron levels are low, this marker is especially helpful for identifying iron deficiency during pregnancy [31]. A study by Carriaga et al. reported that pregnant women’s sTfR levels elevated from 5.36 mg/L around 28–32 weeks to 6.21 mg/L around 36–40 weeks of gestation [32]. However, in this study, we found a decrease in the sTfR levels from 7.55 µg/mL at 12–16 weeks to 5.79 µg/mL at 26–30 weeks. The possible reason for the decrease in the sTfR level may be related to how the body might utilize and store iron during the second and third trimesters. A hemodilution effect, enhanced overall nutrition or good adherence for the oral iron supplementation can lead to improved body iron status.

In this study, maternal iron parameters increased progressively from the second to early third trimester but subsequently stabilized or showed a slight decline toward term. This trend reflects physiological adaptations in iron homeostasis during late pregnancy. Iron requirements rise steeply in the third trimester, reaching nearly 7 mg/day, to support rapid fetal growth, placental expansion and maternal erythropoiesis. During this period, the fetus acquires nearly 80% of its total iron endowment, resulting in preferential transfer across the placenta despite ongoing maternal supplementation. As a consequence, maternal circulating indices often plateau when fetal demands surpass the capacity of absorption and mobilization from maternal reserves. Ferritin concentrations typically decrease in late gestation due to depletion of maternal storage iron and the hemodilutional effect of plasma volume expansion, while transferrin saturation may stabilize or decline because of increased utilization for hemoglobin synthesis. Furthermore, maternal hepcidin is physiologically suppressed throughout pregnancy, promoting iron transfer but preventing further rises in circulating indices. Thus, the late gestational stabilization or mild decline in iron parameters represents physiological redistribution, underscoring the need for trimester-specific monitoring and tailored interventions to optimize maternal and neonatal outcomes [33].

### 4.2. Trimester Specific Strategy and Counselling Support

The temporal variation in maternal iron indices underscores the need for trimester-specific strategies in anemia management. In early pregnancy, when iron demands remain modest, routine screening and initiation of prophylactic iron–folic acid supplementation are essential to prevent latent deficiency. During the second trimester, with rapid expansion of maternal red cell mass and placental growth, dose adjustment and close biochemical monitoring are warranted, and parenteral iron may be considered in women with poor response or intolerance to oral therapy. In the third trimester, as fetal iron requirements peak and maternal indices decline, strategies should focus on sustaining fetal supply and preventing worsening anemia through intensified monitoring, reinforcement of adherence and selective intravenous iron use. Antenatal sessions that combine dietary guidance, education on side effects and their management, and reinforcement of compliance across trimesters significantly enhance maternal awareness and uptake. Engagement of community health workers and family support further promotes adherence, especially in rural settings. Counselling therefore functions not only as an educational tool but also as a behavioural strategy that addresses practical barriers, ensuring sustained compliance and maximizing the effectiveness of supplementation programmes.

### 4.3. Strengths and Limitations

This study included multiple hematological and biochemical markers to assess the response to oral iron supplementation, thus providing a thorough understanding of iron status during pregnancy. Our study found statistically significant changes in key hematological indices including hemoglobin, hematocrit, MCV, MCH, MCHC and sTfR with a high adherence rate to IFA supplementation, confirming its efficacy. Our findings extend the existing body of evidence on oral iron supplementation in pregnancy by providing a detailed longitudinal assessment of hematologic and biochemical markers across gestation in moderately anemic women. While previous studies have largely established the efficacy of oral iron through pre–post comparisons, few have delineated the trimester-specific trajectories of iron indices during supplementation. Moreover, the inclusion of reticulocyte hemoglobin and immature reticulocyte fraction as sensitive indices of erythropoietic activity adds novel insights into the early and real-time responsiveness of bone marrow to oral iron therapy, parameters that are not routinely considered in antenatal programmes. This study was conducted on moderately anemic participants who were iron-deficient in a single location, and the heterogeneity in adherence and responsiveness to iron supplementation observed might limit the generalizability of the findings to a larger population and to other geographic areas. Additionally, this study did not follow up with participants until 6 weeks postpartum to assess the long-term impact of iron supplementation on maternal and infant health.

## 5. Conclusions

This study demonstrated progressive improvement in iron and red cell parameters among moderately anemic pregnant women receiving daily IFA supplementation, with significant gains from the second to early third trimester and stabilization towards term. These findings highlight the need for trimester-specific monitoring of iron status, early initiation of antenatal care and strengthened adherence counselling. Implementing these strategies may improve maternal hematologic status and may contribute to better pregnancy and neonatal outcomes.

## Figures and Tables

**Table 1:** Baseline ands-demographic characteristics (*n* = 315).

Variables	*n* (%)/Median (Q1, Q3)
Maternal Age (years)	
18–25	231 (73.3)
26–30	60 (19.0)
≥31	24 (7.6)
**Maternal Hb (at recruitment)**	9.36 (8.55, 9.74)
**BMI**	
Underweight	106 (33.7)
Normal weight	183 (58.1)
Overweight	22 (7.0)
Obesity	4 (1.3)
**Blood Pressure**	
Systolic Blood Pressure (mmHg)	101 (94,109)
Diastolic Blood Pressure (mmHg)	64 (60,70)
**Parity**	
Nulliparous	134 (42.5)
Primiparous	90 (28.6)
Multiparous	91 (28.9)
**Diet**	
Vegetarian	23 (7.3)
Lacto-vegetarian	47 (14.9)
Ovo-vegetarian	54 (17.1)
Mixed diet	191 (60.6)

**Table 2:** Reasons for not taking iron–folic acid tablets among pregnant women.

Reason	Iron Tablets *n* = 17 (%)	Folic Acid Tablets *n* = 11 (%)
Forgot to take tablets	2 (0.63)	2 (0.63)
Tablets made me feel sick	14 (4.44)	8 (2.54)
Tablets got lost	0 (0)	0 (0)
Did not understand the instructions for taking tablet	1 (0.32)	1 (0.32)

**Table 3:** Comparison of maternal red cell indices in early second trimester and 26–30 weeks of gestational age (*n* = 315).

Parameters	Time Points		*t*-Test/Z Statistic	*p*-Value
	12–16 Weeks GA	26–30 Weeks GA		
RBC (106 cells/µL) ^#^	4.19 ± 0.42	4.05 ± 0.44	7.39	<0.001 *
MCV (fL) ^#^	72.16 ± 7.90	83.47 ± 7.65	26.95	<0.001 *
MCH (pg) ^#^	22.44 ± 3.01	26.77 ± 3.08	24.41	<0.001 *
MCHC (g/dL) ^#^	31.03 ± 1.14	32.01 ± 1.21	12.26	<0.001 *
RDW (%) ^¥^	17 (15.5, 18.10)	16.9 (15.1, 19.7)	3.04	<0.001 *
HCT (%) ^#^	29.93 ± 2.87	33.71 ± 3.69	15.02	<0.001 *
Reticulocyte Hb (pg) ^#^	23.30 ± 3.03	27.84 ± 3.83	18.40	<0.001 *
Immature Reticulocyte Fraction (%) ^¥^	6.90 (4.30, 9.50)	7.30 (4.3, 11.0)	3.59	<0.001 *

^#^ Paired *t*-test (Mean ± SD); ^¥^ Wilcoxon matched-pairs signed-rank test (Median (Q1, Q3)); * Statistically significant.

## Data Availability

The original contributions presented in this study are included in this article. Further inquiries can be directed to the corresponding author.
